# Seeing Beyond Diseases and Disorders: Symptom Complexes as Manifestations of Mental Constituents

**DOI:** 10.3389/fpsyt.2018.00681

**Published:** 2018-12-11

**Authors:** Maurício V. Daker

**Affiliations:** Departamento de Saúde Mental, Universidade Federal de Minas Gerais—UFMG, Belo Horizonte, Brazil

**Keywords:** symptom complexes, dimensional diagnosis, unitary psychosis, personality, psychopathology, history of psychiatry, philosophy and psychiatry, sciences of mind

## Abstract

Many psychopathologists have approached symptom complexes without prejudging them as physical deficits or diseases, an approach suitable to connections with normal mind, to a broad dimensional and anthropological view of mental disorders. It contrasts with the prevailing orientation in psychiatry toward the medical model of delimited diseases. Discussions of this order centered on symptom complexes gained special prominence in psychiatry between the early 20th century through Alfred Hoche and World War II through Carl Schneider. Their works, in addition to the work of other authors of that period, are considered. The late Kraepelin conceded the possibility that affective and schizophrenic manifestations do not represent disease processes but rather represent areas of human personality. Seeing mind or persons is a paradigmatic different perspective than seeing diseases. Re-emerge in this comprehensive or integrationist context the notion of unitary psychosis and philosophical questions as the mind-body problem; as background there was a process metaphysics. The possibility of human experience in a phenomenological sense is considered, and a matrix of symptom or function complexes is related to it. Examples of past unitary models of mental disorders with their neurophysiologic explanations are given, as well as an analogy to current biological aspects of the endogenous in chronobiology. The question or hypothesis arises whether mental symptom complexes are manifestations of mind constituents or functions that make human experience and mind possible. The present work is a conceptual analysis that indicates a positive answer to this question. The expectation is to emphasize the perspectives of investigation in psychopathology and sciences of mind fostered by this view of symptom complexes.

## Introduction

“Of the madman, we all have a little,” is a folk psychological assertion that should hold scientific interest and be considered more seriously. The mainstream notion of disease in psychiatry, aiming to discover specific mental disorders and to demarcate normality from abnormality, may give an artificial impression of simple, detached things, rather than intricately connected factors and causes. There is no unquestionable scientific evidence, however, that major mental diseases or disorders are clearly divided from normal mind, or even divided among themselves. An epistemological clash is embedded here, among the hybrid condition “constituted by the blending of components arising from disparate sources of knowledge ranging from the biological to the semantic in its widest sense” (1), or that “symptoms and signs cannot be properly understood or identified apart from an appreciation of the nature of consciousness or subjectivity, which in turn cannot be treated as a collection of thing-like, mutually independent objects” (2).

Mainstream psychiatry, however, was not always concerned with a strict notion of disease or was epistemologically naive in this way. Apart from the psychodynamic and the phenomenological—anthropological orientations, the history of psychiatry tells of many psychopathologists who saw close relations or a continuum through mental disorders and normality. This was the case in the last century between the 1910s and World War II, a period in which symptom complexes were conceived and investigated more neutrally in relation to possible mental diseases (3).

At that time, the optimism and certainties concerning science in its positivistic contours were shaken at their foundations, as shown by the paradigmatic shifts in physics from atomism to quantum and relativity theories. In psychology and psychiatry, the prime foundation-shaking forces were the phenomenological movement and psychoanalysis. Psychiatry was questioning the previously believed linearity in mental diseases among causes, anatomic pathological findings, and clinical manifestations, as stated in Kraepelin's treatises. In 1920, however, Kraepelin conceded the possibility that “the affective and the schizophrenic manifestation forms of insanity do not represent, in themselves, the expression of certain disease processes, but merely reveal those areas of our personality in which they take place” [(4), p. 27].

Seeing diseases and seeing “areas of our personality” or “a man's character” [(5), p. 549] are totally different conceptions or perspectives. The latter view led those psychopathologists to approach more comprehensively the psychopathological manifestations, to the point of asserting deep relations to normal mind.

## Static Specific Diseases or Categories of Discrete Substances vs. Dynamic Whole Persons

“Hard” science of positivistic contours aims to isolate the studied object from possible external interferences, including from subjective factors. The isolated object or part is investigated and sometimes elucidated in its own limits, as a reduced unit, which can well be summed to other units in a mosaic form. If we know how the pieces of a clock work, we will know how the clock works, a reasoning usually applied also to cells, organs and the organism in medicine. The problem is how far such reasoning is suitable to investigate persons or mind. In the clock model we deal with more tangible facts and concrete concepts; with persons and mind, with more flowing processes and abstract concepts. There a more static approach is feasible, whereas here a dynamic approach seems to be demanded, including an ongoing dialectic process between the parts and the whole, which can be more relevant than a simple sum of isolated parts. Similarly, it can be said that atoms, molecules, genes, or neurons act not simply mechanistically or deterministically to originate mind in a bottom-up one-way road. Top-down concerns are needed, thinking here in the man-made world that implies agency and beliefs, historical process and culture, and intersubjectivity—that means in cognitive sciences a “top-top process” (6), i.e., a collective top interacting with an individual top. All is integrated in a dynamic, comprehensive and non-reducible real unit: the whole person.

Embedded in these discussions are the ancient contrasting views of static delimited substances vs. dynamic flowing processes, which can be tracked in philosophy as far as Parmenides and Heraclitus. Concerning mental disorders, the categorial vs. dimensional diagnosis or classification reflects these contrasting or complementary views. Importantly, as mentioned by Kendell [(7), p. 136]: “Those whose interest is primarily in the nature of the relationship between different syndromes, and between illness and normality, will probably prefer to think in dimensional terms.” Dimensions are prone to dynamic systems in a complex network of internal and external interactions, rather than being based on independent and comparatively discrete substances as are categories. Taken in the sense of a discrete substance, in a dogmatic uncritical one-sided fashion, the medical idea of disease can impose a constraint upon advancement in psychiatry, since isolated units of diseases are self-sufficient and not suitable for dynamic considerations about interrelationships among mental disorders and normal mind. However, if we take the idea of categories less rigidly, e.g., as patterns or prototypes, it allows thinking psychopathology as a matter of degree as opposed to a matter of kind, as shared liability factors conceptualized as continuous dimensions of personality that confer risk for the development of psychopathology (8).

The relation of mental symptom complexes to the whole person indeed allows or, as we will see, demands a more dynamic view in psychiatry that encompasses both the normal mind and the world. Here we are in tune, as alluded above when referring to dynamic flowing processes, with a process metaphysics, which would be more adequate to many scientific fields or approaches than the classical Western metaphysics based on static substances or on an assembly of them (9). Probably not a mere coincidence, the process philosophy was facing a revival in the early 20th century, or turned itself a more distinct branch of philosophy, with Alfred North Whitehead, Henri Bergson, Martin Heidegger, and many others (9).

## Symptom Complexes, Person, and Mind

According to Hoche (5), it is due to the occurrence of the special, constant, and largely merging forms of reactions, i.e., psychic dispositions such as hysteric, hypochondriac, neurasthenic, suspicious, querulant, etc., the pressing indication that in the normal psyche certain symptom associations or complexes lie preformed. “In part they make up what we call a person's character. In part, in the case of special illness-inducing influences, determine how the morbid deviant form of reaction of the personality occurs” [(5), p. 549]. Hoche thinks that the same applies word by word to the most named mental disorders, such as the symptom complexes melancholia, mania, chronic paranoia. These symptom complexes are therefore observed in mental disorders that appear to be only a strengthening of certain morbid personal dispositions, or they can also be triggered by organic processes, here with a secondary significance [(5), p. 549]. Hoche is striking against the idea that these symptom complexes might be like disease entities, as conceived in general medicine, comparing the investigation of disease entities in psychiatry to a hopeless and exhausting hunt for ghosts:

It underlies all these strenuous efforts [to find disease entities], the indestructible belief that it should be possible also in psychiatry to find particularly defined, pure, single forms of disease, a belief that again and again is nourished from the analogy to somatic medicine, without thinking that the nature of the relationships between symptom and anatomical basis, as they are here and there, cannot be compared at all with each other [(5), p. 542].

The psychic, following Hoche, represents an entirely new category, obeying its own laws, not commensurable with material processes, like music is not comparable to the musical instrument. In the same sense, we can say that biology produces the instrument and culture writes the music.

In being more malleable than the previous, rigid Kraepelinean view of disease entity, or because of it, the new approach entails an inevitable anthropological perspective in psychopathology, that is, entails normal aspects of the mind and the person. The late Kraepelin was already contemporaneous to or was anticipating interest by many psychopathologists in the personality of the patient, as would become prominent in mainstream psychiatry of that time through Ernst Kretschmer's work (he achieved a nomination in 1929 to the Nobel Prize), among many others, also those of phenomenological orientation like Minkowski, who emphasized the whole living personality in psychiatry. As far as for dementia praecox or schizophrenia, Kretschmer's work, also Eugen Bleuler's, ended up perceiving, in Oswald Bumke's words, “nothing but a morbid condensation of normal mental reactions” (10), originated in the characteristic personality of the schizophrenic patients. Except in cases of schizophrenia, Bumke himself saw a smooth transition between the endogenous or functional diseases and the manifestations of healthy mental life (11).

Ferdinand Kehrer, commenting on Hoche, welcomed “the displacement of an elementary psychology strange to life through a matter of lived personality within psychiatry” [(12), p. 433], which is also a warning against rigid schematizing. According to him, “preformed” or “to lie ready” should be understood as disposition, which manifests either in the permanent mental constitution or in the readiness for mental disorders. When confronted with a somatic or a psychogenic involvement or etiology, the dispositions act as a “pathogenetic intermediate constituent” between that etiology and the manifestation. Kehrer mentions here Karl Bonhoeffer, who fomented the discussion on the typical syndromic reaction that mediate different etiologies.

Karl Jaspers had already considered the intermediate position of symptom complexes, in this case between the elementary manifestations or symptoms and the disease units, and he advised that “they should be studied in themselves, regardless of disease unit and disease processes, to investigate the regularity and needful togetherness that exist in them” [(13), p. 268]. According to Jaspers [(14), p. 491], Carl Schneider placed symptom complexes at the epicenter of psychiatry. Schneider diverged clearly from Kraepelin's conception of disease entities in psychiatry with its linear correlations among etiology, anatomopathological findings, and clinical manifestation. Importantly, Schneider's “symptom associations” (some of them were empirically investigated by him, concerning schizophrenia) are intimately related to normal “function associations,” to the point of saying that the cluster of symptoms in the symptom complexes are already present in normal mind and, for differentiation, are here better named function associations [(15), p. 142]. It is emphasized that the identified associations are not static constructions in the sense of a rigid categorization but rather expressions of an always-fluid process of life with dynamic effects and changeable responsiveness [(15), p. 194]. All reflects his broad concept of “biological” (3).

## Human Experience and Reality: The Role of Symptom/Function Complexes

Philosophers have much to say about how human beings access or constitute reality. Following Kraus (16), we can trace a deepening in the subject from Kant to Heidegger, going through Husserl. These philosophers also established the foundations of the phenomenological—anthropological psychiatry. Kant's transcendental categories of mind or consciousness are inherent to and make possible the human experience. Husserl emphasized the new understanding of consciousness as reality-constituting through its intentional acts, mingling subject and object. Heidegger sees a fusion among being human and world by means of the presence or existence, which is prior to consciousness and to the self. Heidegger's term for this presence or existence is *Dasein*, which as human “being-in-the-world,” in a constant interplay of existence and the historical world or sedimented meanings, stands open to other beings and to itself, constituting one's world and the self through endowing of meaning. Still following Kraus, the existentials, which are fundamental structures of *Dasein*, act in this sense as Kant's categories: “The existential, or to say it better, the existential a priori, give meaning to and thereby constitute the world of anybody (in the present discussion, the patient). As such, they are one's matrix of possible experience” [(16) p. 101]. Altered “existentials a priori” or pre-objective being interferes with the constitution of the most fundamental ontological components of reality, as space, time, causality, and objecthood, making possible many kinds of strange self-disorders, hallucinations, and delusions (16, 17), that is, making possible or constituting a different reality. Kraus also asserts the need for a dynamic reasoning here: “For psychopathology it is important that being on this level, as these notions already indicate, is by no means something static, but has to be understood as a process of happening” [(15), p. 102].

Maiese (18) considers that one of the aims of phenomenology has been to outline invariant formal structures of consciousness, as the categories or existentials mentioned above, that serve as necessary constraints on human experience. This author points out that a systematic analysis of these structures is central to an enactive conceptualization of subjectivity and self-consciousness and describes five necessary structures of consciousness: spatial, temporal, egocentric (an inner source-point), intentional, and conative affective. They are physically grounded in the endogenous processes and self-organizing neurobiological dynamics of our living animal bodies and constitute a natural matrix of a basic sensorimotor subjectivity to be understood as a system of causal-dynamic relations [(18), p. 9–13].

The point in the present work is whether, on the psychopathology side, the above considerations on symptom complexes would also allow thinking about a matrix that makes human experience or the human mind possible. “Symptom” or “function complexes” also occur within anyone and would act to constitute one's world or the human world. The disconnectedness, the perception becoming “an object of noetic awareness” or a “disembodied spirit” (19) in schizophrenia might not be entirely disadvantageous: It could be necessary for abstraction or for a detachment of an immediate bond with things and the world. It could be an important feature for imagination or creativity, which is found in the relatives of patients, comprising for example divergent thinking and originality (20). This capacity of loose or broad associative thinking and flexibility in schizophrenia might be contrasted or somehow balanced with the over-systematized and rigid pole of paranoia, which in fact also seems to have advantageous aspects—e.g., religions or normal necessary beliefs. Both schizophrenia and paranoia would be intercrossed with the poles of depression and mania responsible for affective attunement, as well as for over-involvement in social situation and commitment to social norms in depression. The pole of obsession expresses part of the rigidity of paranoia and the over-attunement of depression, while hysteria or dissociation might express a mingling of schizophrenic disconnectedness and aspects of mania. Relevant here is that, as well as an ordering of symptoms in each symptom complex, there would be an ordering among the symptom complexes, recalling the tradition in psychopathology of the unitary psychosis (21, 22); ahead are described the unitary psychosis or continuum of symptom complexes by Guislain and Griesinger]. Such dynamic and integrative ordering among the symptom/function complexes is what is meant when thinking about them acting as a matrix in human experience and mind, either in normal as in the considered psychopathological conditions.

Taken in this dynamic configuration, regarding the primary mental disorders, there would be no specific anomalies properly or necessary deficits, but a systemic modification or disequilibrium. As in the ancient Hippocratic humoral theory, whereby humors are not anomalous by themselves, function complexes are necessary to normality when in balance. In other words, there would be a disorder and restriction of quality of life (whether or not it is called “disease” or “symptoms”) due to a substantial unbalance of essential human qualities or functions—of the “intermediate constituent” or functional matrix. In such a disproportional and condensed way, it is possible to understand, with Carl Schneider, that symptom complexes can be a clue to the knowledge of normal function associations [(15), p. 237]—that is, the unbalanced pathological disorder highlights the more fluid and thus less accessible connections of normal functions. Again, we need to think about a dynamic process to be able to see possible interrelations in all directions among the complexes, whether deviant (unbalanced) or not. Better to think here in terms of forces, as in electromagnetism, whose physics influenced the process metaphysics and vice versa, and where in a certain sense everything is everywhere at all times (23, 24), rather than thinking on an interaction of discrete detachable substances or parts.

It is probably that the phenomenological-anthropological psychiatry and the theory of symptom complexes are confluent in important aspects, also because they emerged concurrently. Likewise, process philosophy was concurrent, as already mentioned, seemingly underpinning both or being close related to them. Associating these approaches should be promising to (re)opening perspectives in psychopathology in an embracing spectrum: philosophy and medicine, mind and body. For example, it can be conceptualized that the endogenous symptom/function complexes interact early in life with the living body sensations, with human environment and intersubjectivity, from where categories or existentials could emerge, a process that turns out to be like a procedural memory and, hence, a priori or pre-reflexive. Much of that could be suitable to neuroscientific or neurobiological investigations [(25, 26), p. 311–37].

## Unitary Psychosis and the Endogenous in a Neuroscientific Approach: Past and Future

As a branch of medicine, psychiatry has always been concerned with anatomophysiological thinking, usually in the search of specific biological markers. But also, due to the own dynamicity of the mind and its psychopathologies, psychiatry has, since its beginnings, often offered more holistic or systemic conceptions of mental disorders in relation to other branches of medicine. Examples are Joseph Guislain's and Wilhelm Griesinger's unitary views of mental disorders.

Guislain sustained in the first half of the 19th century one general modus of evolution for all mental disorders: from manifestations of sensibility or *Phrenalgie* (*lypérophrenie* or melancholia), followed by reactions (*hyperphrénie* or mania, *paraphrénie* or folly, *hyperplexie* or ecstasy, *hyperspasmie* or convulsion, *idéosynchysie* or delusion and mental aberrance, and *anacoluthie* or mental disintegration), up to an end in annihilation or destruction (*noasthénie* or dementia). The disorders described in each of these three groups would usually mix one after another or at the same time, in such way that he believed to be able to describe over 100 disorders. As Kraepelin (1920) explains it, there would be a great variety of disorders according to the location and intensity of brain impairments. Guislain influenced Wilhelm Griesinger, who among others propagated the usual evolution of mental disorders through continuous stages: melancholia, mania, psychic weakness, and dementia, each of them with typical symptom complexes, and manifold mixed states between them (27).

The clinical observations corroborating those usual stages were convincing enough: think also about general paralysis, which evolves from affective to dementia stages, and was around 30% of the hospitalized patients. Yet Griesinger added an appealing physiological explanation, indeed a “neuropsychophysiological” explanation, to it. He began from an analogy of the normal functioning of the spinal cord with the brain, which would be an evolutional prolongation of the former. The charge of energy or tonus in spinal cord (responsible for the muscle tonus) becomes psychic tonus and (mostly unconscious) idea associations in the brain, and instead of motricity there are aspirations, which are voluntary, with liberty of action, or impulsive. The movement of the psychic tonus and of the ideas results in emotions and affects. Normal mental reflection or deep thinking corresponds to a normal physiological slow-down of the afferent, central and efferent pathways flux. When the flux becomes hampering, however, it results in the state of melancholia, with its manifestation of hypersensitivity to the dammed afferent stimuli, psychic pain, sluggish thinking, no action, and so on. On the other hand, mania emerges from a convulsion-like reaction in spinal cord, bringing immediate action after the afferent stimuli—mania is energy to volition and impulses. All these affective manifestations can return to normality, but if there is damage to this physiology—think of a rubber band that is so stretched and loosened that loses its elasticity or tonus—it results in the secondary clinical manifestations, which are chronic: “a wreckage of a boat after the storm,” in Griesinger's words [(27), p. 324], a loss of the psychic tonus and of idea associations—and also of liberty. This “wreckage” can sink further down into dementia.

In his later life, Griesinger assumed a more neuropathological and less physiological and dynamic view of mental disorders, since it became accepted that paranoia would begin without the prior affective stages. This development challenged his physiological conception that had presupposed the presence of primary affective stages (apart from rash brain damage). The neuropathological wave in psychiatry is well known, for there was a time of eminent neuropsychiatrists. Conversely, Karl Kahlbaum, Kraepelin, and colleagues believed that psychiatry should first do its homework by describing the psychiatric diseases; otherwise the neuroanatomists or neuropsychiatrists would fall on their faces, as it indeed happened on the side of psychiatry.

The problem is that the new described diseases, though laborious and judiciously described, were not as such proven, at least in the strict sense of disease as they were supposed to be. Then, the theory of symptom complexes emerged with its connectedness to personality and openness to normal aspects of mind, as we saw above.

This theory was virtually abandoned after World War II, and the strict medical model of disease soon prevailed again in psychiatry, after the psychodynamic and the phenomenological-anthropological waves (3). It is difficult to say to what neuroscientific approach it would correspond nowadays. Here an analogy with the concept of endogenous in chronobiology might be useful in some respects. Decades ago, it was demonstrated that living beings have their own endogenous or internal biological rhythms, which are being elucidated from anatomical structures, genetics, and molecules. The endogenous rhythms are supposed to couple with the external rhythms or *Zeitgebers* (“time givers”), facilitating or making possible the adaptation and survival of living beings. It is vital to adapt to night and day, and in many cases also to tides, moon cycles, and seasons—that is, to the geophysical cycles. What in chronobiology are called endogenous oscillators originated in physics: They synchronize their rhythms as two pendulums attached to a beam tend to synchronize their movements. Such dynamic rhythmicity of several integrated oscillators makes the organism malleable or adaptable to different internal and external conditions (28).

It would similarly succeed with the matrix from which the symptom complexes originate: Past biological conditions in phylogeny concerning the relations between the living being, as well as the human being, with the environment would have been incorporated genetically, leading to the endogenous preformed function complexes that make adaptation and survival possible. Of course, the relation of living beings with geophysical rhythms is much simpler than within the context of mind and its relation to historical, social, cultural, or linguistic aspects. Hereby, the *Zeitgebers* would be better thought as *Sinngebers* (“sense givers” or “meaning givers”). The relation between the historical world as *Sinngeber* and the person, self or existence could be considered here, provided that endogeny is conceived in the latter. William Stern's process of “introception” might also involve such relationship between historical process and person, for it indicates a personal act through which values can be taken up from the cultural milieu by individuals and appropriated or embraced as their own during personality development [(29), p. 329]. Living beings, including human beings, must be prepared, predisposed, or “preformed” for the situations with which they will interact in the world to which they pertain. The synchronization of function complexes with the world or with *Sinngebers* would be a process of “structural coupling” (30), “a phase in the co-temporaneous development of two systems (e.g., organism and environment) where mutual dynamic dependencies unfold across system boundaries” (9). In other words, the multivariate genomic level intersects with the ways in which genetic effects are contingent on environmental moderators (8). The subject of this work is an attempt to glimpse *how* this relation between biology and environment might proceed in the realm of mind.

## Conclusions

Following Jaspers in his introduction to *General Psychopathology*, psychopathology faces a wide, impenetrable continent of which we have knowledge only from its edges, one of body and one of meaning. Indeed, it seems that in the present work we are traveling through a shadowy region in between. Dealing with psychiatry and philosophy, which profoundly influences psychopathology, or dealing with mental disorders, exposes this situation. As a branch of medicine, psychiatry is in relation to philosophy more suitable to the edge of the body, whereas philosophy is prone to meanings without delving much into anatomophysiological or neurobiological concerns. Both should be complementary in the investigation of our continent. It is the case that phenomenological psychopathology already offers accurate descriptions and original conceptions that can aid the neurosciences, but some concepts as existentials or Kantian categories, which make the human experience possible, seem difficult to relate to biology. Psychopathology might contribute to an advance in this puzzle with its theory of symptom or function complexes coming from medicine, even though the knowledge of their anatomophysiological or neuroscientific basis are still incipient. Better it is to say that psychopathology and the function complexes might be indispensable in regarding the mediation between organism, its world, and mind—or, between biology and culture. Symptom/function complexes are manifestations or basic chords of delicate instruments, making possible the human mind symphony.

## Author Contributions

The author confirms being the sole contributor of this work and has approved it for publication.

### Conflict of Interest Statement

The author declares that the research was conducted in the absence of any commercial or financial relationships that could be construed as a potential conflict of interest.
